# Community disruption in small biogenic habitats: A coastal invader overcomes habitat complexity to alter community structure

**DOI:** 10.1371/journal.pone.0241116

**Published:** 2020-10-26

**Authors:** Andrey V. Malyshev, Paula Tummon Flynn, Ruth Cox, Cristian Duarte, Pedro A. Quijón

**Affiliations:** 1 Institute of Botany and Landscape Ecology, Greifswald University, Greifswald, Germany; 2 Department of Biology, University of Prince Edward Island, Charlottetown, PE, Canada; 3 Department of Health Management, Atlantic Veterinary College, University of Prince Edward Island, Charlottetown, PE, Canada; 4 Facultad de Ciencias de la Vida, Universidad Andres Bello, Santiago, Chile; 5 Centro de Investigación Marina Quintay, CIMARQ, Universidad Andrés Bello, Santiago, Chile; University of Sydney, AUSTRALIA

## Abstract

Non-indigenous species are often identified as threats to native species and communities. Yet, the mechanisms that enable many of these invaders to thrive and alter their newly invaded habitats are still not fully understood. This applies to habitats such as widespread sedimentary shorelines characterized by the presence of scattered biogenic clumps of blue mussels (*Mytilus edulis*) structurally more complex than bare sediments. In Atlantic Canada, some of these shorelines are numerically dominated by native mud crabs (*Dyspanopeus sayi*) but have been gradually invaded by the European green crab (*Carcinus maenas*). This study describes between-habitat (mussel clump vs. bare sediment) differences in density and diversity of invertebrates. It also tests the impact of juvenile green crabs in comparison to native mud crabs using two approaches: First, measuring habitat-related differences in these crabs’ feeding rates on a common prey (soft-shell clams, *Mya arenaria*). Second, measuring their influence on invertebrate communities associated with mussel clumps. The results show that mussel clumps hold higher invertebrate density and diversity than surrounding sedimentary bottoms. In the laboratory, the feeding rates of native mud crabs were dependent on the type of habitat (sand flat > mussel clump), whereas those of green crabs were significantly higher and unrelated to the habitat in which predation occurred. In field experiments, juvenile green crabs were also the only predators that changed community structure in the mussel clump habitat. These results indicate that green crabs can cause a significant impact on native species and communities. Moreover, they suggest that the ability of this species to overcome the refuge provided by complex biogenic habitats for prey may represent an unexplored mechanism to explain this invader’s expansion here and elsewhere.

## Introduction

The spread of marine non-indigenous species has been a recurrent topic in the ecological literature [1–4]. Nonetheless, the mechanisms mediating their success remain poorly understood and their impacts are still far from predictable [5–7]. Most studies addressing the influence of non-indigenous predators have focused on consumption rates, prey preferences [8, 9] and intra-guild interactions [10, 11]. As a result, the ultimate impact of invaders on species assembly and diversity has often received less attention than the study of more direct effects on individual (often commercially valuable) species [12–14]. However, a growing number of studies have shown that non-indigenous species have community-wide and food web effects that should not be disregarded, particularly when these invaders are predators [4, 15, 16]. In habitats such as marine soft-bottoms, epibenthic predators can have an unparalleled impact in regulating community structure and function [17–20]. However, when these relatively smooth and featureless sediments are associated with structurally complex biogenic habitats such as eelgrass, mussel or oyster beds [21], the impact of predators becomes less clear [22–25].

Clumps of mussels create three-dimensional substrate of hierarchical, irregularly shaped patches [21, 26–28] and represent an interesting system to explore the impact of new predators in the context of habitat-related interactions. The structural and functional characteristics of mussels can influence patterns of biodiversity and play a role in structuring soft-bottom communities. Their physical presence creates spatial refuges from predation and environmental stress, provides substrate for epifauna, stabilizes sediments, and alters water flow [25, 29–30]. They are also able to selectively filter suspended particles from the water column, including larvae of benthic species [31] and deposit organic material within the bed [32]. Previous studies comparing invertebrate communities inside and outside of mussel beds have reported different species composition, including greater diversity and/or abundance, in communities associated with mussel patches in soft-bottoms [33–38] and hard-bottoms [39–43].

Mussel clumps and beds are ubiquitous features of coastal habitats around the world and are a prominent feature of Atlantic Canadian sedimentary shorelines. In the Atlantic region, these patchy biogenic habitats are numerically dominated by small native mud crabs (*Dyspanopeus sayi*) but have also been colonized by juvenile stages of the non-indigenous European green crab (*Carcinus maenas*) [44–46]. Changes in predatory guild composition and abundance following the arrival of non-indigenous species can have cascading effects on communities [4, 15]. Green crabs, in particular, have been negatively associated with native crustacean and bivalve populations [46–49]. They have also been found to be responsible for habitat disruption, including local-scale changes (e.g., invertebrate density in feeding pits; [50]) and much larger-scale disruptions of biogenic habitats, for example the loss of large eelgrass beds in Nova Scotia and Newfoundland [51, 52] and formerly extensive soft-bottom mussel beds in the Gulf of Maine [53–57].

The first goal of this study was to document the contribution of blue mussels (*Mytilus edulis*) to the local-scale abundance and diversity of intertidal invertebrate communities in soft-bottom habitats in Atlantic Canada. We hypothesized that the biogenic structural complexity introduced by mussels alters community diversity and/or abundance relative to that on bare sediments. The second goal of this study was to assess the impact of juvenile green crabs in comparison to the impact of the similar-sized native mud crab. We hypothesized that crab predation on an individual species is altered in habitats with more complexity (mussel clumps vs. bare sand) and that native and non-indigenous crab predators differ in their impacts on infaunal density and community composition in complex habitats. We tested these hypotheses by measuring habitat-mediated feeding rates on a common type of prey (juvenile soft-shell clams, *Mya arenaria*) in lab enclosure experiments, and then assessing predator influence on community composition and abundance in mussel clumps through field enclosure experiments.

## Material and methods

### Study area and predator densities

**Fig 1:**
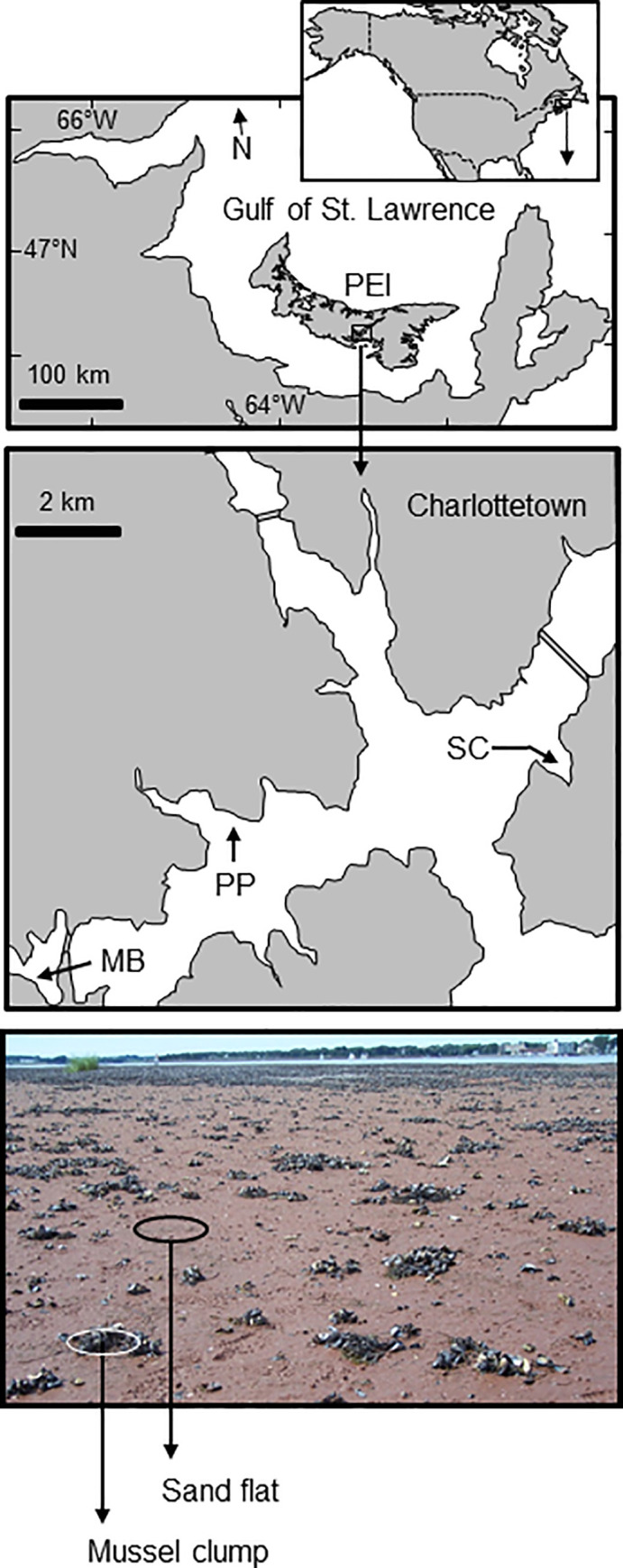
Map outlining Prince Edward Island (PEI) in Atlantic Canada, with the approximate location of the Hillsborough estuarine system (arrow). The insert shows the location of Meadows Bank (MB), Primrose Point (PP), and Stewart Cove (SC). The photograph illustrates mussel clumps scattered within sedimentary bottoms in the field sites.

Three intertidal sites with extensive sandy flats and abundant mussel clumps were sampled in the Hillsborough estuarine system, southern Prince Edward Island (Fig 1): Primrose Point (46’12’35”N, 6311’01”W), Meadow Bank (46’11’19”N, 63’14’22”) and Stewart Cove (46’13’23”N, 63’07’09”W). The three areas are located within 5 km from each other within the distribution range of green crabs [44, 45] and mud crabs [46]. These three sites are characterized by similar tide ranges and intertidal zones with gentle slopes associated with eelgrass beds and prominent salt marsh formations. *In-situ* densities of mud crabs in the past decade (S1 Fig) have consistently been higher in mussel clumps (~1.0–5.5 crabs 625 cm^-2^; hereafter 1.0–5.5 crabs quadrat^-1^) than on nearby sandy sediments (~0–0.3 crabs quadrat^-1^) (see field survey methodology below). Those measurements (2006, 2007 and 2014) established a gradual decline in the average number of mud crabs over time, a trend also reported for the southern Gulf of St. Lawrence [46] using time-series data of mud crab abundance measured with beach seine samples from multiple locations. In contrast, abundances of non-indigenous green crabs in the study area and the region have gradually increased since their establishment [46], although they are highly variable and strongly site-dependent (see Pickering et al. 2017 [9] and Poirier et al. 2017a [45] for data on temporal changes in density of green crabs).

### Field survey

To characterize the local composition and density of invertebrates associated with mussel clumps (i.e., discrete patches of blue mussels and shells; Fig 1) and surrounding sandy sediments, a one-time survey was conducted to collect samples from one of the sites referred to above (Stewart Cove) during low spring tides of June and July 2007. Eight mussel clumps, approximately 20 cm in diameter, were randomly selected and sampled using 25x25 cm stainless steel quadrats (625 cm^2^) inserted ~5 cm into the substrate. Clumped mussels, shells, sediments, and debris were collected using spatulas and then washed through 2 mm sieves. The choice of sieve size was based on feasibility for processing and handling. While we acknowledge a risk of underestimating the diversity of small species, a large and diverse number of invertebrates were collected in the samples. Each sample of a mussel clump was paired with a sample collected from bare sediments (those lacking mussel clumps or shells), from the immediate vicinity (0.5–1.0 m away). The material retained in the sieves was preserved in 70% ethanol, sorted and identified to the species level using standard identification keys for the region [58, 59].

### Laboratory enclosure experiment: Habitat-mediated crab feeding rates

Based on the results of the field survey (see Results), juvenile soft-shell clams (*Mya arenaria*) were selected as prey to compare feeding rates of native and non-indigenous crabs as it was one of the most abundant species in both mussel clumps and bare sediments. This species is a component of the diet of mud crabs and green crabs [60] and juvenile clams (6–10 mm Shell Length, SL) are known to be consumed by both species in the study area. Previous experiments have also used this species to compare crab feeding rates. Soft-shell clams were collected manually from nearby sediments in Stewart Cove. Adult mud crabs (25–35 mm CW) were compared to juvenile green crabs of a similar size range because predator (crab) size can influence feeding trial outcomes [9, 61]. Juvenile green crabs were considered the logical counterpart of native mud crabs because they exploit the same prey items and habitats as consistent occupants of intertidal mussel, oyster and algae clumps (P. Quijón Pers. Obs.; [62]). All the crabs for experiments were collected from Belvedere Pond, located approximately 2 km NE from Stewart Cove, within the same estuarine system. Only unharmed (without missing claws or legs) male crabs of each species were used to avoid potential biases associated with sex and condition [63]. The crabs were starved for 48 h prior to their use in any experiment to avoid potential biases associated with crab’s prior diet [64].

Twenty juvenile soft-shell clams were placed into each of ten 19.8 L glass tanks containing seawater (~25 ppt; 18°C) and a bottom 3 cm layer of cleaned sandy sediments from the study area. The clams were allowed to acclimate and bury themselves in the sand for approximately 1 h before one individual mud crab or green crab was introduced. Trials were run once for 24 h with the top and sides of each tank covered with dark paper to avoid crab escape and to reduce light exposure and potential visual distractions [65]. In addition to trials with bare sediments, additional trials (mussel clump mimic treatment) were conducted in identical tanks in which a 3 cm layer of mussel shells (considered representative of the thickness of mussel layers in the field) was added on top of the sediment to resemble the physical structure of a mussel clump. While shells cannot fully mimic live mussels [see 56, 57, 66] they were collected from the same field site, had a similar size range (~15–35 mm SL), and have been used in the past as habitat mimics in other studies [46]. At the end of each trial the predator was removed from the tank and the number of remaining soft-shell clams was counted to estimate percent mortality of prey (or % of clams eaten). Experiments were initiated and terminated at about the same time of the day (late morning) A few trials in which crabs showed signs of molting were not considered for any analyses and conducted again with different crabs. To avoid potential learning after repeated trials, each crab was used only once [65].

### Field enclosure experiment: Crab effects on mussel communities

Mussel clumps of approximately the same size (~20 cm diameter) were randomly selected from the mid-low intertidal of Stewart Cove to conduct a 1-wk field enclosure experiment in 2008. These discrete clumps were 1–2 m apart and embedded in bare unstructured sandy sediments (Fig 1). Mussel clumps located at least 10 m apart were identified (tagged with straws) and randomly assigned to the following treatments: green crab enclosure (GC), mud crab enclosure (MC), cage with no crabs added (control cage; CC) and un-manipulated control (clump with no cage or crabs added; NC). The experiment included a total of eight replicates per treatment. Three mud crabs or three green crabs were placed in each of the enclosure cages (MC and GC treatments, respectively). These crabs were collected from the same location and had the same size range as those used in the laboratory experiments. Their density within the cages was approximately half the highest mean density observed in the field surveys conducted in 2006, 2007 and 2014 (up to a mean of 5.5 crabs clump^-1^; see details in S1 Fig) and was consistent with repeated observations of mussel clumps in the study area and other areas nearby during those years. Cages were circular, (35 cm in diameter and 30 cm high) bucket-shaped cylinders built with ~0.5 cm wire mesh around a metal rod frame. The cages were inserted upside-down (open bottom, covered top) approximately 5 cm into the sediment and anchored with long plastic pegs. To avoid unnecessary disturbance of sediments and invertebrates associated with the mussel clumps, only visible mud crabs or green crabs were manually removed from the clumps before cage placement.

For the duration of the experiment, the cages were checked daily during low tide for potential stranding and accumulation of eelgrass (*Zostera marina*), rockweed (*Fucus serratus*) or Irish moss (*Chondrus crispus*) or build-up of fine sediments (i.e., to visually assess for potential cage-artefacts; [67]). Eelgrass or seaweed stranding were observed on the cage sides in only a few cases and were removed immediately. There was no visual evidence of sediment accumulation within or outside the cages. At the end of the experiment, cages were carefully opened and the mussel clumps examined until the three crabs that had been introduced were found, recorded and removed. Mud crabs were released on site whereas green crabs were retained and returned to the laboratory (none of the green crabs or mud crabs escaped the cages and no additional crabs were found in any of the cages). After crab removal, a quadrat was inserted immediately around the mussel clump, and samples were collected and processed. Blue mussels were in general much larger (> 2cm SL) and less abundant than other invertebrates and therefore were not considered plausible prey for the small predators used in this experiment. In addition, blue mussels were not considered part of the infaunal communities described below.

### Data analyses

Invertebrate species richness and density recorded in bare sediment and mussel clumps in the field were compared using paired t-tests. Density comparisons included total number of invertebrates and the density of polychaetes, bivalves, and four of the most abundant species. Data on species composition and density were also analyzed to assess community similarity between habitats using the Bray-Curtis index and the program Plymouth Routines In Multivariate Ecological Research (PRIMER 6). Data were fourth-root transformed to proportionally reduce the influence of the most abundant species. Visual differences among treatments were inspected using a non-metric Multi-Dimensional Scaling (MDS) plot. Analysis of Similarity (ANOSIM) was then used to assess whether or not the differences between habitats were significant [68]. The SIMPER routine was also applied to identify the species that contributed the most to the dissimilarity between habitats.

Comparisons of the number of soft-shell clams eaten in the laboratory (percent mortality) by either mud crabs or green crabs in bare sediments or mussel clump habitats were analyzed with a two-way ANOVA. Independent factors included the predator (green crab vs. mud crab) and the habitat (mussel clump vs. sand flat); both factors were considered fixed.

For the field enclosure experiment, species richness and densities (total and density of bivalves, polychaetes, and the four most abundant species) recorded in the four treatments (GC, MC, CC and NC) were compared using one-way ANOVAs. In cases where the ANOVAs detected significant differences, Holm-Sidak *a posteriori* tests were used to identify individual pairwise differences between treatments. Assumptions of normality (Shapiro-Wilk test) and equal variance were checked in all instances. Due to violations to the first assumption, a log_(n+1)_ transformation was applied to the data except in two cases: species richness (raw data) and density of *H*. *filiformis* (sq-root transformation). These analyses were conducted using Minitab® routines. Untransformed data on species composition and density were also analyzed to assess community similarity among treatments using PRIMER 6 routines (MDS, ANOSIM and SIMPER) as described for the field survey.

### Permits, animal care and ethics

Permit to access field sites in Prince Edward Island were obtained from the Department of Fisheries and Oceans Canada–Gulf Division (seasonal permit to collect organisms for scientific purposes). All applicable institutional (ACC-UPEI) and national guidelines for the care and use of animals were followed. This article does not contain any studies with human participants.

## Results

### Field survey

**Fig 2:**
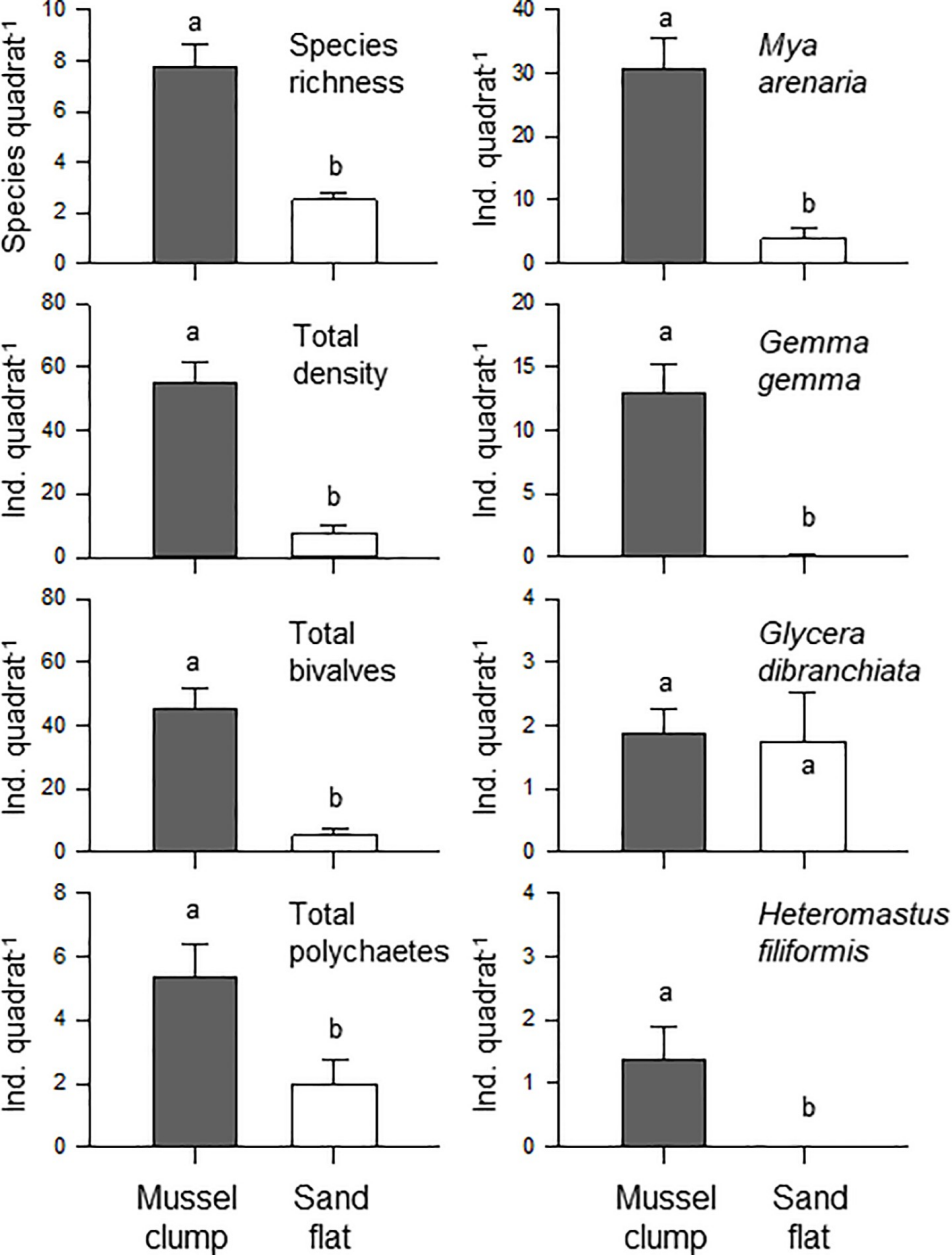
Mean (+S.E.) species richness and density for overall communities, bivalves (except blue mussels) and polychaetes, in addition to four of the most abundant species collected in mussel clumps and sandy flat sediments (filled and open bars, respectively). Different letters above the bars stand for significant differences between habitats (paired t-test p<0.05).

Species composition and average density are summarized in S1 Table. The average number of species was three times higher in sediments associated with mussel clumps than in those associated with surrounding bare sands (7.75 and 2.5 species quadrat^-1^, respectively; paired t-test p = 0.001; Fig 2; Table 1). Total densities, total density of bivalves and polychaetes, and densities of soft-shell clams (*Mya arenaria*), gem clams (*Gemma gemma*) and the spionid polychaete *Heteromastus filiformis*, were on average between 3 and 7 times higher in the mussel clumps than in surrounding sandy sediments (all differences were significant, paired-t test p≤0.036; Fig 2; Table 1). In contrast, the polychaete *Glycera dibranchiata* was found at similar average densities (1.88 and 1.75 individuals quadrat^-1^ in mussel clumps and sand flats, respectively; paired t-test p = 0.857; Fig 2; Table 1).

**Table 1:** Results of paired t-tests comparing infaunal species richness and densities between habitats (mussel clumps versus sand flats) from a field survey conducted at Stewart Cove. N = 8 and DF = 7 in all the analyses. Significant p-values are in bold.

Dependent variable	Paired t-test value	p-value
Species richness	5.274	**0.001**
Total density	6.041	**<0.001**
Bivalves density	5.214	**0.001**
Polychaetes density	3.160	**0.016**
*Mya arenaria* density	4.594	**0.003**
*Gemma gemma* density	5.823	**<0.001**
*Glycera dibranchiate* density	0.188	0.857
*Heteromastus filiformis* density	2.582	**0.036**

**Fig 3:**
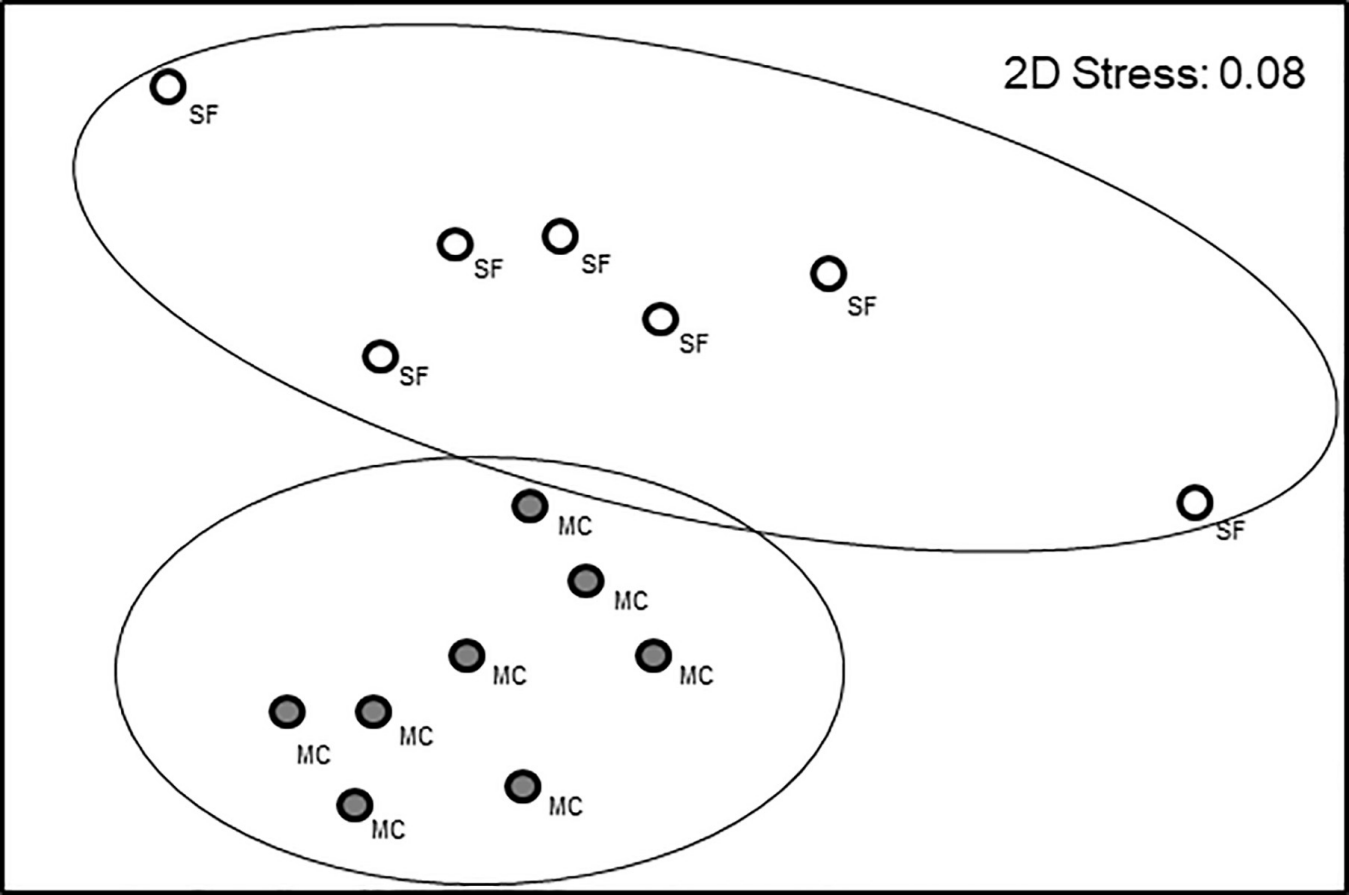
MDS plot illustrating Bray-Curtis community similarity among samples associated with mussel clumps (MC; filled circles) and sandy flat sediments (SF; open circles). Lines encircling samples apart from all the other samples are based on significant differences detected by ANOSIM (p<0.05).

The nMDS plot (Fig 3) identified a strong difference in composition and abundance of communities associated with mussel clumps and bare sediments. The nMDS plot was also a good representation of the variation among samples (2D stress value = 0.08) and showed that samples associated with mussel clumps segregated apart from samples associated with bare sediments. ANOSIM tests confirmed that between-habitat differences were significant (Global R = 0.588, p = 0.002). The average level of dissimilarity between habitats was 65.8% and the most important species driving the dissimilarity between habitats were the bivalves *Gemma gemma* and *Mya arenaria* and the isopod *Jaera marina* (See detailed results in S2 Table).

### Laboratory enclosure: Habitat-mediated crab feeding rates

**Fig 4:**
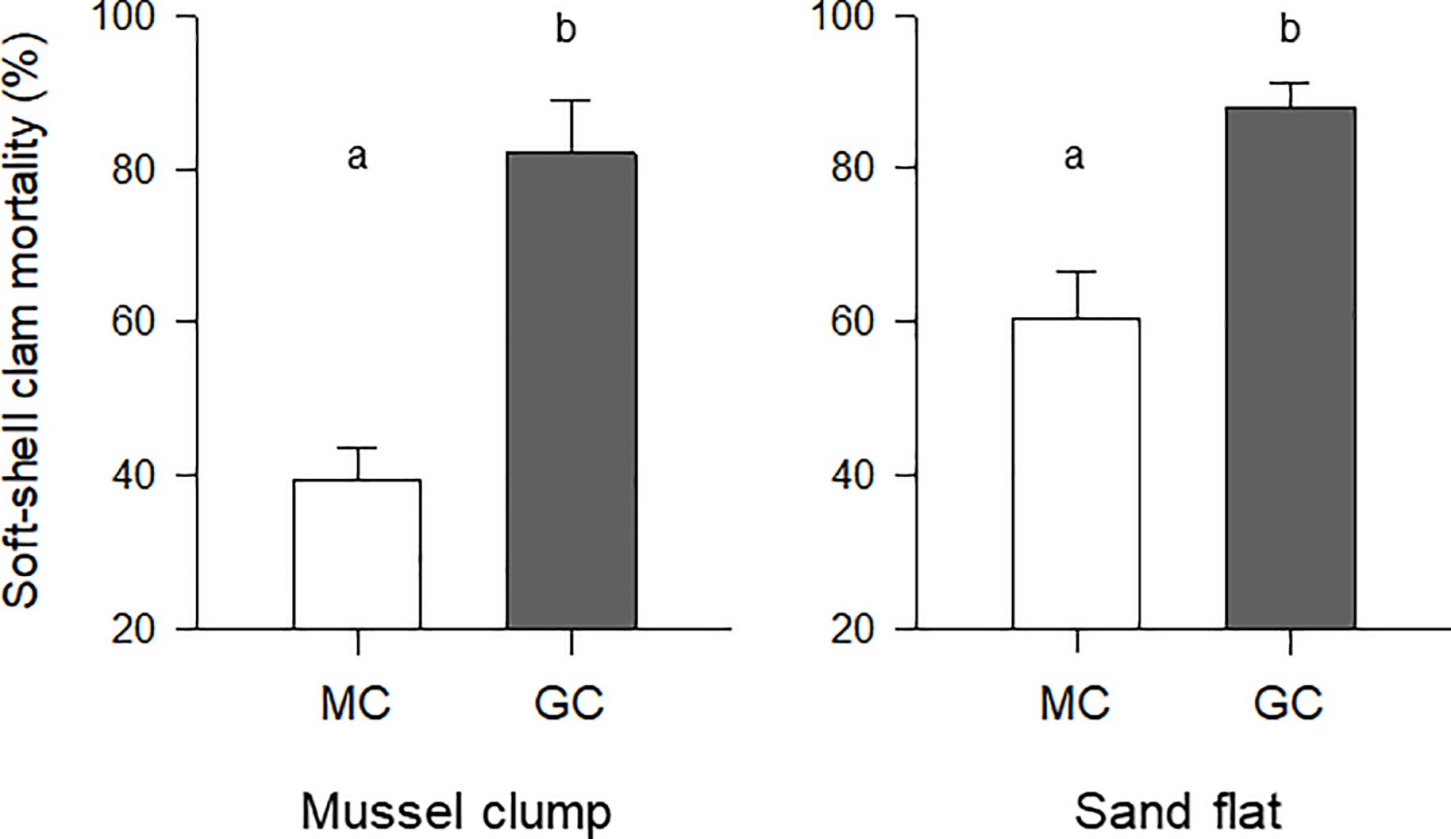
Mean (+S.E.) soft-shell clam percent mortality resulting from predation by mud crabs (MC, open bars) and juvenile green crabs (GC, filled bars with a green crab at top) in 24 h laboratory trials. The experiments were conducted in tanks with mimics of mussel clumps and sandy flats. Different letters above the bars stand for significant differences between predators (two-way ANOVA p<0.05). Between-habitat differences were also found but are not included for clarity.

Predator identity and habitat each had a significant influence on the percent mortality of juvenile soft-shell clams (two-way ANOVA p<0.001 and p = 0.025, respectively), whereas their interaction was not significant (two-way ANOVA p = 0.230). Green crabs consumed significantly more juvenile soft-shell clams than mud crabs, eating ~60% and ~38% more clams than mud crabs in mussel clumps and sand flats, respectively (Fig 4). When comparing feeding rates in relation to habitat, mud crabs consumed substantially fewer soft-shell clams in mussel clumps than in the sand flat (average clam percent mortality was ~40% and 60%, respectively). In contrast, juvenile green crabs consumed only marginally different amounts of soft-shell clams: average mortality of ~82% and 88% in mussel clumps and sand flats, respectively; Fig 4).

### Field enclosure: Crab effects on mussel clump communities

**Fig 5:**
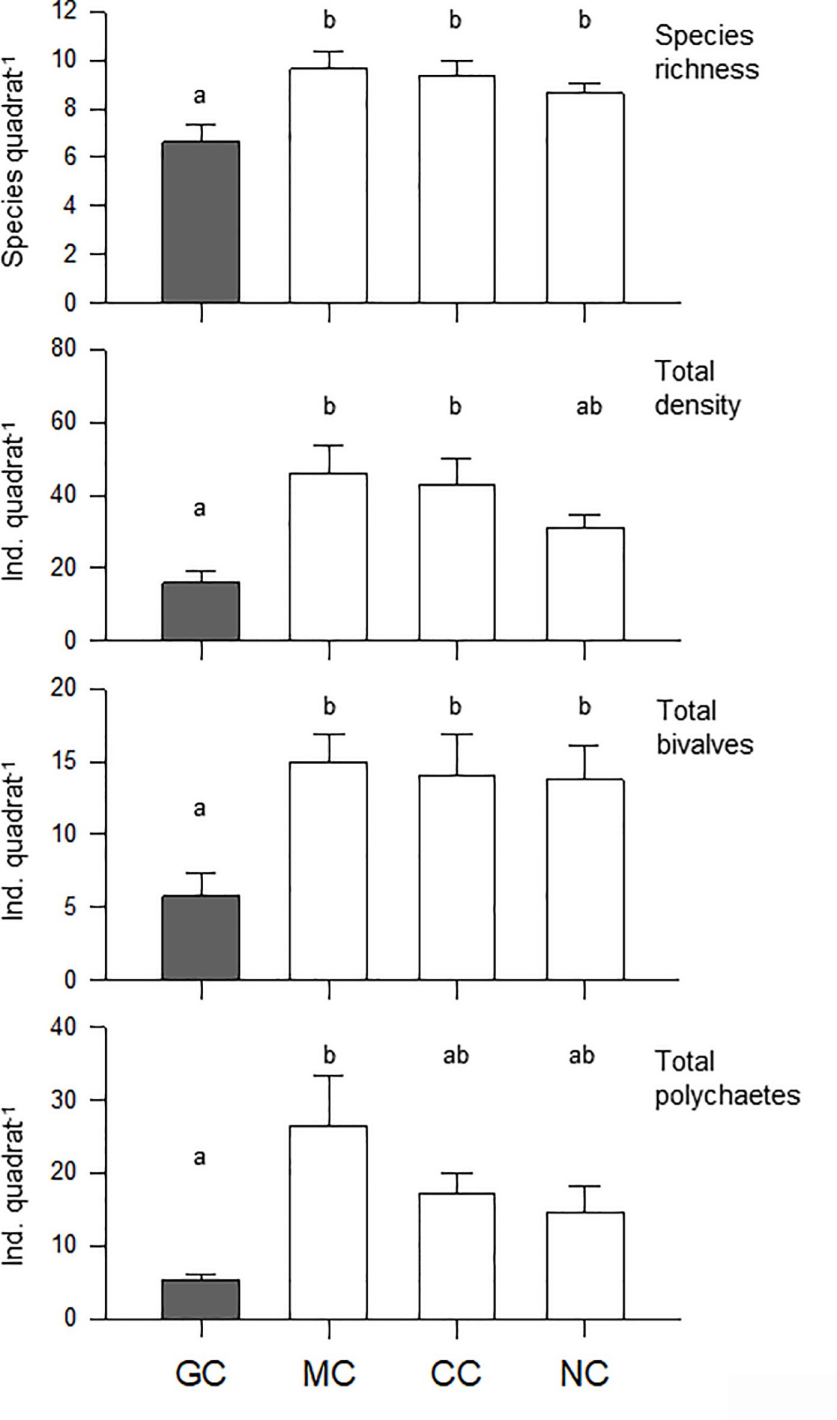
Mean (+S.E.) species richness, total density and density of bivalves and polychaetes for each of the four treatments included in the field enclosure experiment. GC: green crab enclosure (filled bars); MC: mud crab enclosure; CC: clump in a control cage; NC: no cage or clump without a cage. Different letters above the bars stand for significant differences among treatments based on Holm-Sidak post-hoc comparisons (p<0.05).

**Fig 6:**
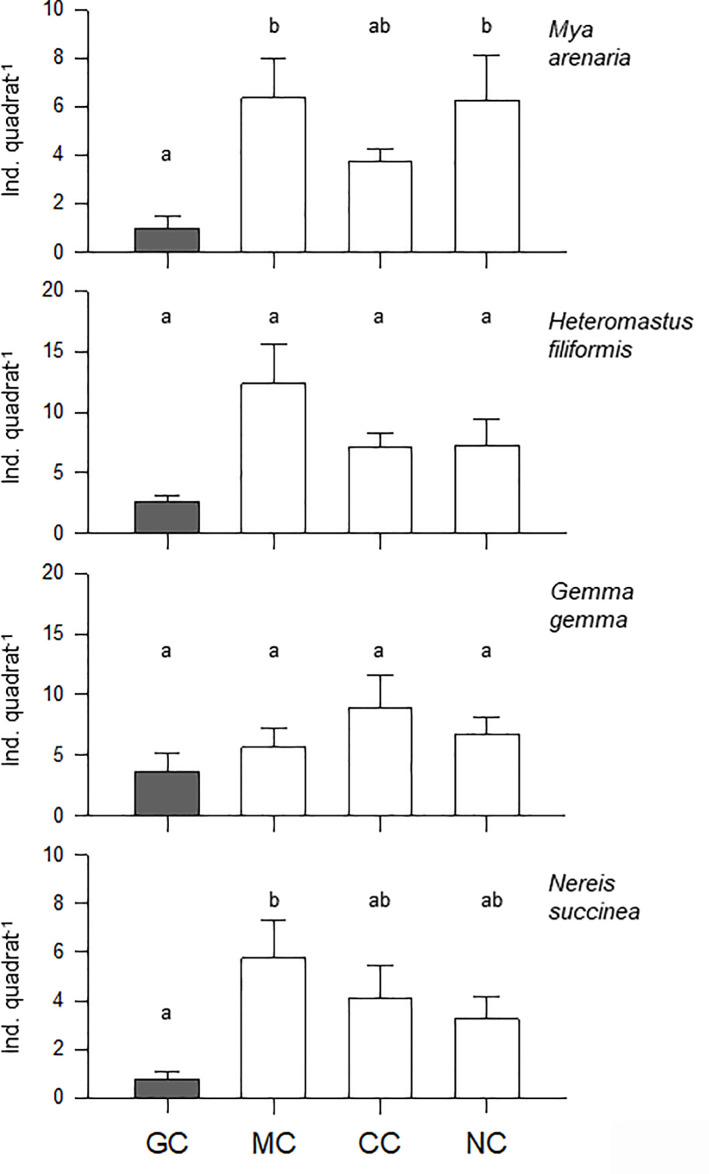
Mean (+S.E.) density of the four most abundant species for each of the four treatments included in the field enclosure experiment. Other details as in Fig 5.

Species composition and average density are summarized in S3 Table. The average number of species in the mussel clumps was significantly lower in green crab enclosures (~6.6 species quadrat^-1^) than in any other treatment (mud crab enclosure, caged and uncaged controls; one-way ANOVAs p = 0.009; Fig 5, Table 2). In general, similar results were obtained when quantifying total densities and the density of bivalves (one-way ANOVA p<0.001 and p = 0.003, respectively). The total density of polychaetes was significantly different only when comparing green crab and mud crab enclosures (one-way ANOVA p<0.001; Fig 5). Regarding the density of numerically dominant species (Fig 6), mussel clumps exposed to green crab predation generally exhibited significantly lower averages of soft-shell clams and *Nereis succinea* (one-way ANOVA p<0.001 and p = 0.003, respectively; Table 2). However, the density of small gem clams (*Gemma gemma*) and *Heteromastus filiformis* did not differ among treatments (p = 0.083 and p = 0.089, respectively; Fig 6; Table 2).

**Table 2:** Results of one-way ANOVAs comparing infaunal species richness and densities from the four treatments of the field enclosure experiment: green crab enclosure (GC), mud crab enclosure (MC), control cage (CC), and no cage (NC). N = 8 and DF = 3,28 in all the analyses. For simplicity, only Mean Squares (MS), F- and p-values are presented. Significant p-values are in bold. Holm-Sidak post-hoc comparisons are illustrated in Figs 5 and 6.

Dependent variable	ANOVA components		
	MS	F	p
Species richness	14.792	4.733	**0.009**
Total density	0.343	12.283	**<0.001**
Bivalve density	0.385	6.097	**0.003**
Polychaetes density	0.475	8.129	**<0.001**
*Mya arenaria* density	0.560	9.028	**<0.001**
*Heteromastus filiformis* density	3.575	2.466	0.083
*Gemma gemma* density	0.265	2.396	0.089
*Nereis succinea* density	0.459	5.931	**0.003**

**Fig 7:**
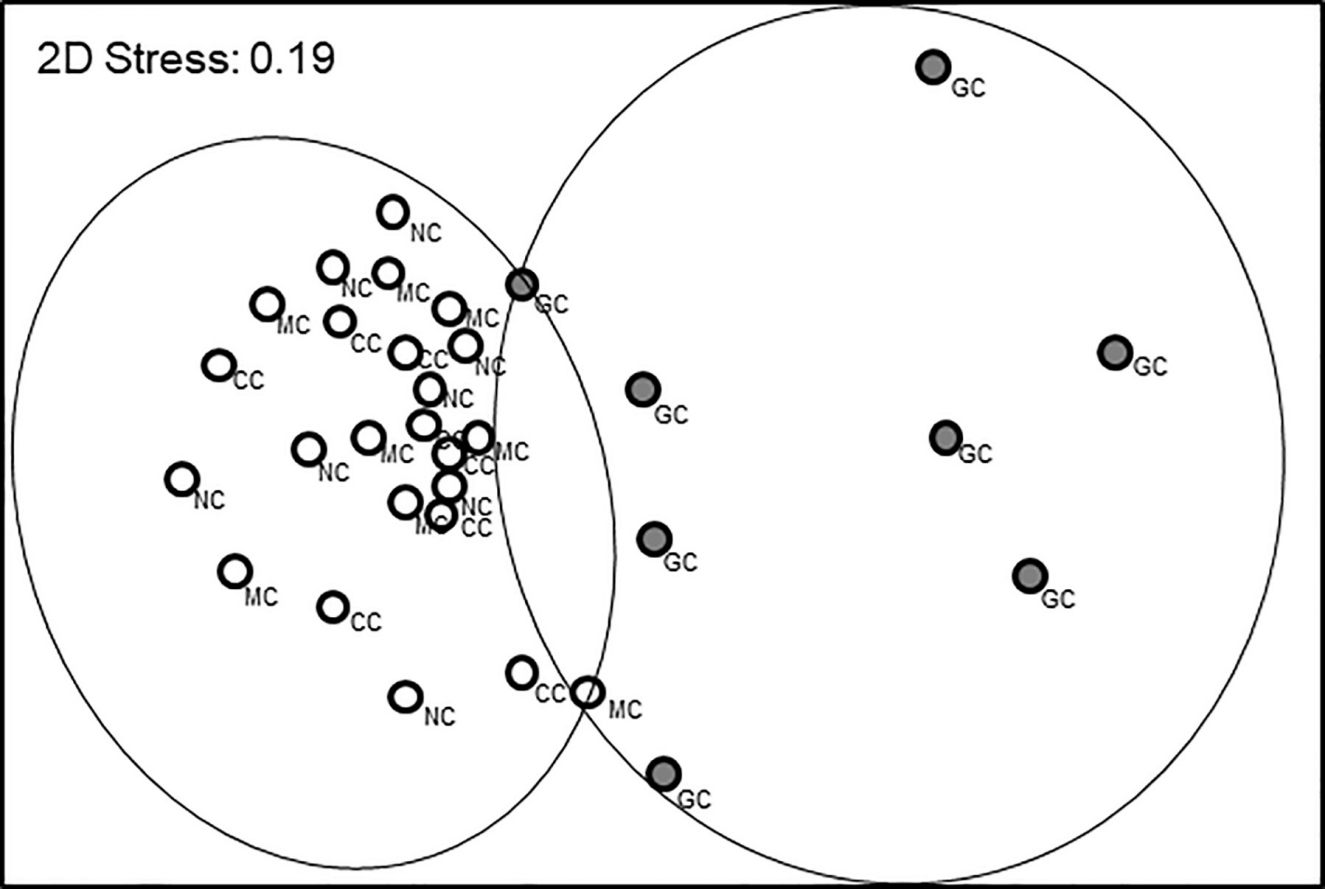
MDS plot illustrating Bray-Curtis community similarity among samples associated with the four treatments of the field trial. GC (filled symbols): green crab enclosures; MC: mud crab enclosures; CC: control cage; NC: no cage or clump with no cage. Lines encircling green crab enclosure samples apart from all the other samples are based on significant differences detected by ANOSIM (p<0.05).

The nMDS plot (Fig 7) identified a strong effect of green crabs on community structure and a fairly good representation of the variation among samples (2D stress value = 0.19). The samples associated with green crab enclosures formed a distinct cluster separated from the samples from all other treatments (mud crab enclosures, and caged and un-caged controls). ANOSIM tests confirmed that the green crab enclosure was the only treatment significantly different from others (Global R = 0.169, p = 0.003). P-values for pair-wise comparisons between green crab enclosure and the other treatments were all significant (p = 0.001–0.006), whereas p-values for pair-wise comparisons among the remaining three treatments were all non-significant (p = 0.322–0.714). The average level of dissimilarity between green crab enclosures and the three other treatments ranged between 68.3 and 72.30%. The most important species driving the dissimilarity between habitats were the polychaete *Heteromastus filiformis* and the bivalves *Gemma gemma* and *Mya arenaria* (See S4 Table for details).

## Discussion

Green crabs can be effective predators in unstructured sediments such as sandy and muddy tidal flats [47, 50, 69]. The results gathered here suggest that they can also be effective predators in habitats that typically reduce or preclude the effects of epibenthic predation [24, 70]. Juvenile green crabs were more effective predators than native mud crabs, and they demonstrated an ability to catch and consume prey in both unstructured and structured habitats. These results reinforce the potential for green crabs to have negative community-wide effects [16]. Moreover, they suggest that the ability of this species to overcome the refuge provided by complex biogenic habitats could represent a fairly unexplored mechanism to explain this invader’s expansion here and elsewhere. If this argument is applicable to other non-indigenous predators, it may well represent an additional facilitation mechanism for successful invasions [2, 7], more specifically for invasions by predators. This argument fits in the broader examination of the relative effects of native and non-indigenous predators on prey [71, 72]. A review of the literature found that in comparison to their native counterparts, non-indigenous predators were generally more detrimental to terrestrial and avian vertebrates [71] and prey associated with terrestrial or freshwater habitats, but not always with marine habitats [72]. Our study of invertebrate predators and prey adds an interesting case to that theoretical debate.

### Field survey and habitat differences

In comparison to surrounding unstructured sediments, mussel clumps held remarkably high densities and diversity of species. These results support the hypothesis that mussel clumps represent islands of biogenic habitat whose structural complexity increases diversity relative to bare sediment. This is in line with what has been observed in coastal shorelines in Europe [34–36], but not necessarily in eastern North America, where increases in abundance but not in diversity have been reported [33, 37, 38, 56, 57, 73]. It is unclear why diversity results differ between the study area and Maine, USA. Such differences may be the result of different green crab invasion histories [48] or the use of different gear for the sampling of infauna: various quadrats sizes and different sieve mesh sizes. Regardless, these results highlight the importance of biogenic habitats to the full shoreline ecosystem [40, 73]. Understanding the influence of those habitats on the impact of native and non-indigenous predators is relevant, as demonstrated by our laboratory experiments: The analysis of crab feeding rates on a single prey type suggests that native and non-indigenous crabs do not have the same impact and that this impact depends on habitat (see also [92]). Added habitat complexity (mussel shells mimicking biogenic clumps) effectively diminished mud crab feeding rates on soft-shell clams (measured here as clam percent mortality). However, it did little to prevent consistently high feeding rates by juvenile green crabs. Native predators fed on soft-shell clams, but when placed in a more complex habitat, they followed a pattern previously observed elsewhere in e.g., blue crabs consuming scallops and oysters [23, 74]. Although our mussel clump mimics did not fully resemble a clump of live mussels, these mimics reduced clam mortality caused by mud crabs by as much as 40%. This was likely because of a reduction in predator-prey encounter rates resulting from the added difficulty crabs faced finding and handling clams [22, 74].

The clear influence of mussel clump mimics on mud crab feeding rates supports the hypothesis that predation levels are altered (reduced) in habitats with more complexity. Ultimately, habitat complexity may have reduced prey profitability (*sensu* Norberg 1977 and Stephens and Krebs 1986; [75, 76]) for the native predator, although we acknowledge that adding a layer of mussel shells on top of sediments also changed the overall depth of the habitat available to the clams to seek refuge. In contrast, juvenile green crabs consumed significantly more clams than mud crabs did, and they did so regardless of habitat complexity and depth. It is unclear what enables juvenile green crabs to be successful predators in sharply different habitats. However, from prior observations of green crabs digging for prey around roots and shoots of eelgrass (*Zostera marina*) [77], it can be speculated that this ability is related to an unusually broad repertoire of movements allowing them to seek prey in relatively complex habitats. Prior studies have shown that green crabs possess quick learning skills and adaptability to handle new types of prey and to gather food (bivalves) using novel techniques [78, 79]. For prey such as the soft-shell clam, which typically gains refuge from predation within structured habitats such as seaweeds and shellfish clumps and beds [40], the evidence presented here is also concerning from a practical point of view. The arrival of predators well-suited to effectively exploit structured as well as unstructured habitats may entail a new, and potentially substantial, source of mortality for multiple prey. Species of commercial importance, such as the soft-shell clam, make the outcome of these interactions potentially detrimental for coastal growers and harvesters [9, 12, 74] and the industries they support.

### Green crab effects on mussel clump communities

During the field experiment conducted here, the density and diversity of species associated with mussel clumps were altered solely in the presence of the non-indigenous predator. Unlike the native mud crab, green crabs reduced overall diversity and abundance by ~30%. The impact of green crabs in this habitat was surprising as the complexity of the mussel clumps was expected to limit crab foraging abilities [23, 92]. However, the results presented here are consistent with the results from the laboratory experiment and suggest that this non-indigenous predator may thrive in unstructured as well as structured habitats. These results also provide strong support to the hypothesis that native and non-indigenous crab predators differ in their impacts on infaunal communities in complex habitats. The literature on soft-bottom communities contains a wealth of examples where population densities or life history traits (e.g., size or growth rates) have changed following the addition of epibenthic predators [17–20, 80, 81]. However, only a handful of studies have documented changes in the number of species in a community. In the Atlantic region, the native rock crab (*Cancer irroratus*) is one such case [82]. Those authors documented a marked decrease in density and species richness following the enclosure of rock crabs [82]. The changes reported here are not as striking quantitatively, but were measured in habitats (mussel clumps) expected to be less prone to strong predator effects.

The effects of green crabs may not be restricted to those documented here. Gehrels et al. [46] recently showed that adult green crabs also feed on mud crabs, which suggests multiple, perhaps synergistic negative effects (*sensu* Myers 1989 [83]) on this native species. Similarly, competitive interactions or various indirect effects between crabs inhabiting mussel clumps are plausible and cannot be discounted. Due to logistic limitations, the design of our field experiment did not include a fifth treatment where mud crabs and green crabs were confined together. Interactions between these predators may change their individual effects on infauna, perhaps adding predation pressure (additive or non-additive effects) or, more likely, attenuating their overall effects [47]. The examination of intra-guild interactions was beyond the scope of this study, but would likely shed light on some of the results recorded in the clumps that lack a cage (NC or uncaged treatment). As expected, exposure to surrounding predators caused an “intermediate” effect (i.e., between control and strong green crab effects) on total densities and the densities of polychaetes (including *N*. *succinea* and *H*. *filiformis*), but not on species richness of bivalve densities. The latter may suggest limitations associated with the use of cages [84, 85], which normally include accumulation of fine sediments or changes in predator or prey behavior [84, 85]. We consider the accumulation of fine sediments highly unlikely given the short duration of the field trial [69], and the daily monitoring of cages showing no evidence of cage artefacts. The potential for behavioral responses to the presence of e.g., adult green crabs [16, 46] or other species should be explored further. Similarly, interactions between infaunal organisms may also have an effect here and should be assessed. Prior studies suggest that at least some of these species are predatory infauna [69, 86], a functional group including carnivorous polychaetes and nemertines (e.g., *Lineus viridis* in Maine [33]), which likely add further complexity to the trophic role played by the green crab in these habitats.

### Implications

Invasions stand among various other pervasive sources of anthropogenic disturbance of coastal habitats [87–89]. While the changes measured in this study occurred at the scale of small patchy habitats, they must be considered in the context of the continued expansion of this non-indigenous species in the Atlantic region [44, 45] and shoreline regions in most continents. Additional studies should address larger-scale questions such as the ongoing alteration of structured habitats (beds of eelgrass, mussels and oysters) by green crabs, which may result in increased fragmentation and ultimately, a gradual change in the entire shoreline ecosystem. The decline of once extensive mussel beds in the Gulf of Maine has been linked to the green crab invasion as well as climate change, and represents a key example of a non-indigenous species’ ability to influence entire food webs [16] by simultaneously consuming native species while disrupting biogenic habitat. Over time, as little as 2–4% of the original live mussel coverage remains in mussel beds increasingly fragmented into mussel clumps [56, 57]. As the slow recovery of similar habitats elsewhere suggests (e.g., oyster beds and eelgrass beds [51, 90]), such a change has long-term implications for ecosystems [91] and harvesting industries.

The further spread of green crabs is expected to promote changes among individual prey (soft-shell clams, blue mussels and various other shellfish species [9, 12]), intra-guild counterparts such as mud crabs or other crustaceans ([11, 46, 92], this study), and habitat-forming species such as mussels, oysters and eelgrass [51, 78, 93]. While assessing the likelihood of future invasions by this and other species [94], their ability to modify communities associated with structured habitats needs to be seriously considered. Establishing or recovering foundation species and ecosystem engineers that create structured habitats is broadly perceived as a positive measure for the ecology and function of coastal ecosystems [92, 95]. However, in light of the evidence presented here, this may not necessarily protect every native species from aggressive invaders like the green crab.

## Supporting information

S1 Fig (TIF)

S1 Table Species composition and average density (+/- Standard Error) of assemblages associated with mussel clumps and sand flats collected during the field survey. (DOCX)

S2 Table Ranking of species contributing the most to the dissimilarity between habitats (up to a minimum of 60% dissimilarity) in the field survey of mussel clumps and sandy flats. (DOCX)

S3 Table Species composition and average density (+/- Standard Error) of assemblages associated with the four treatments in the field enclosure experiment: Green crab enclosure (GC), mud crab enclosure (MC), control cage (CC) and no cage (NC). (DOCX)

S4 Table Ranking of 5 species contributing the most to the dissimilarity between green crab enclosures (GC) and the other three treatments in the field experiment: mud crab enclosure (MC), Control cage (CC) and no cage (NC), up to a minimum of 60% dissimilarity). Column at the left identifies the comparisons and the average dissimilarity.
(DOCX)
