# Supplementary figures and images for: Community disruption in small biogenic habitats: A coastal invader overcomes habitat complexity to alter community structure
Source: PLoS One. 2020 Oct 26;15(10):e0241116. doi: 10.1371/journal.pone.0241116 (PMC7588051; doi:10.1371/journal.pone.0241116)

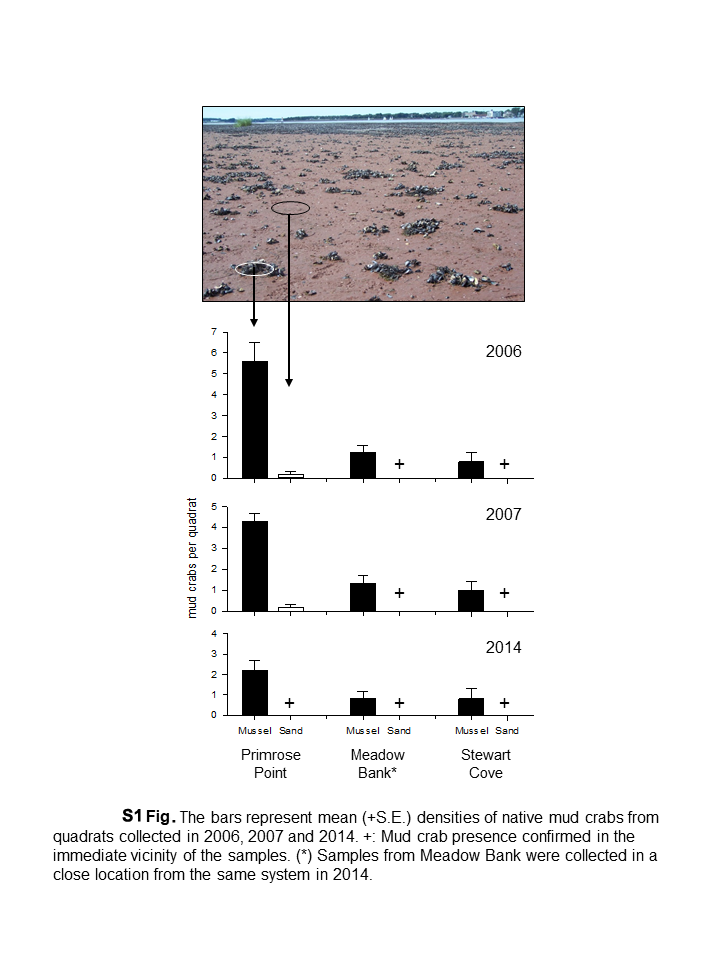

Supplement: S1 Fig — (TIF) [file pone.0241116.s001.tif]
