# Supplementary material for: Community disruption in small biogenic habitats: A coastal invader overcomes habitat complexity to alter community structure
Source: PLoS One. 2020 Oct 26;15(10):e0241116. doi: 10.1371/journal.pone.0241116 (PMC7588051; doi:10.1371/journal.pone.0241116)
Supplement: S1 Table — (DOCX) [file pone.0241116.s002.docx]

**S1 Table. Species composition and average density (+/- Standard Error) of assemblages associated with mussel clumps and sand flats collected during the field survey.**

| **Species** | **Mussel clump density quadrat^-1^** | | **Sand flat density quadrat^-1^** | |
| --- | --- | --- | --- | --- |
|  | **Average** | **Standard Error** | **Average** | **Standard Error** |
| *Hiatella striata* | 1.13 | 0.72 | 0.63 | 0.32 |
| *Mya arenaria* | 30.75 | 4.92 | 3.88 | 1.54 |
| *Mercenaria mercenaria* | 0.00 | 0.00 | 0.63 | 0.42 |
| *Gemma gemma* | 13.00 | 2.28 | 0.13 | 0.13 |
| *Lyonsia hyalina* | 0.13 | 0.13 | 0.13 | 0.13 |
| *Dyspanopeus sayi* | 0.75 | 0.41 | 0.00 | 0.00 |
| *Rhithropanopeus harrisii* | 0.13 | 0.13 | 0.00 | 0.00 |
| *Pagurus acadianus* | 0.13 | 0.13 | 0.38 | 0.26 |
| *Jaera marina* | 2.50 | 1.15 | 0.00 | 0.00 |
| *Polydora quadrilobata* | 0.13 | 0.13 | 0.25 | 0.25 |
| *Harmathoe imbricata* | 0.75 | 0.49 | 0.00 | 0.00 |
| *Heteromastus filiformis* | 1.38 | 0.53 | 0.00 | 0.00 |
| *Nereis diversicolor* | 0.13 | 0.13 | 0.00 | 0.00 |
| *Glycera dibranchiata* | 1.88 | 0.40 | 1.75 | 0.77 |
| *Eteone longa* | 0.13 | 0.13 | 0.00 | 0.00 |
| *Spio filicornis* | 0.25 | 0.16 | 0.00 | 0.00 |
| *Pygospio elegans* | 0.25 | 0.16 | 0.00 | 0.00 |
| *Mediomastus ambiseta* | 0.13 | 0.13 | 0.00 | 0.00 |
| *Ophioglycera gigantea* | 0.13 | 0.13 | 0.00 | 0.00 |
| *Marenzelleria viridis* | 0.13 | 0.13 | 0.00 | 0.00 |
| *Nereis virens* | 0.13 | 0.13 | 0.00 | 0.00 |
| *Belanus arenatus* | 0.13 | 0.13 | 0.00 | 0.00 |
| *Corophium volutator* | 0.75 | 0.41 | 0.00 | 0.00 |
