# Supplementary material for: Community disruption in small biogenic habitats: A coastal invader overcomes habitat complexity to alter community structure
Source: PLoS One. 2020 Oct 26;15(10):e0241116. doi: 10.1371/journal.pone.0241116 (PMC7588051; doi:10.1371/journal.pone.0241116)
Supplement: S2 Table — (DOCX) [file pone.0241116.s003.docx]

**S2 Table. Ranking of species contributing the most to the dissimilarity between habitats (up to a minimum of 60% dissimilarity) in the field survey of mussel clumps and sandy flats.**

| **Species** | **Average densities** | | **Dissimilarity** | |
| --- | --- | --- | --- | --- |
|  | **Mussel clump** | **Sand flat** | **Average (%)** | **Cumulative (%)** |
| *G. gemma* | 1.85 | 0.14 | 13.19 | 20.04 |
| *M.arenaria* | 2.32 | 1.08 | 10.17 | 35.51 |
| *J. marina* | 0.83 | 0 | 5.99 | 44.60 |
| *H. filiformis* | 0.74 | 0 | 5.42 | 52.84 |
| *H. striata* | 0.57 | 0.48 | 4.81 | 60.15 |
