# Supplementary material for: Community disruption in small biogenic habitats: A coastal invader overcomes habitat complexity to alter community structure
Source: PLoS One. 2020 Oct 26;15(10):e0241116. doi: 10.1371/journal.pone.0241116 (PMC7588051; doi:10.1371/journal.pone.0241116)
Supplement: S3 Table — (DOCX) [file pone.0241116.s004.docx]

**S3 Table. Species composition and average density (+/- Standard Error) of assemblages associated with the four treatments in the field enclosure experiment: Green crab enclosure (GC), mud crab enclosure (MC), control cage (CC) and no cage (NC).**

| **Species** | **GC** | | **MC** | | **CC** | | **NC** | |
| --- | --- | --- | --- | --- | --- | --- | --- | --- |
|  | **Average** | **SE** | **Average** | **SE** | **Average** | **SE** | **Average** | **SE** |
| *G. oceanicus* | 2.750 | 2.186 | 2.000 | 1.861 | 4.750 | 13.40 | 0.625 | 0.916 |
| *M. dentata* | 0.250 | 0.164 | 1.000 | 0.189 | 1.500 | 1.690 | 0.875 | 1.356 |
| *A. rubricata* | 1.500 | 0.463 | 1.500 | 0.598 | 4.375 | 8.895 | 1.000 | 2.070 |
| *C. insidiosum* | 0.375 | 0.375 | 0.000 | 0.000 | 0.750 | 2.121 | 0.000 | 0.000 |
| *Unid. Amph* | 0.000 | 0.000 | 0.125 | 0.125 | 0.250 | 0.707 | 0.000 | 0.000 |
| *M. arenaria* | 1.000 | 0.463 | 6.375 | 1.603 | 3.750 | 1.488 | 6.250 | 5.285 |
| *G. gemma* | 3.625 | 1.523 | 5.625 | 1.535 | 8.875 | 7.586 | 6.750 | 3.808 |
| *M. mercenaria* | 0.500 | 0.267 | 0.875 | 0.398 | 1.000 | 1.195 | 0.250 | 0.463 |
| *S. solidissima* | 0.125 | 0.125 | 0.000 | 0.000 | 0.125 | 0.354 | 0.250 | 0.463 |
| *H. striata* | 0.500 | 0.189 | 1.875 | 1.394 | 0.250 | 0.463 | 0.125 | 0.354 |
| *P. pholandii* | 0.000 | 0.000 | 0.125 | 0.125 | 0.000 | 0.000 | 0.000 | 0.000 |
| *A. islandica* | 0.000 | 0.000 | 0.000 | 0.000 | 0.000 | 0.000 | 0.125 | 0.354 |
| *M. balthica* | 0.000 | 0.000 | 0.125 | 0.125 | 0.000 | 0.000 | 0.000 | 0.000 |
| *I. balthica* | 0.000 | 0.000 | 0.000 | 0.000 | 0.000 | 0.000 | 0.250 | 0.463 |
| *N. succinea* | 0.750 | 0.313 | 5.750 | 1.578 | 3.875 | 3.980 | 3.000 | 2.507 |
| *G. dibranchiate* | 0.125 | 0.125 | 2.375 | 0.532 | 1.500 | 0.926 | 1.000 | 1.069 |
| *H. extenuata* | 0.125 | 0.125 | 0.000 | 0.000 | 0.000 | 0.000 | 0.000 | 0.000 |
| *H. imbricate* | 0.125 | 0.125 | 0.125 | 0.125 | 0.000 | 0.000 | 0.000 | 0.000 |
| *H. filicormis* | 2.625 | 0.498 | 12.40 | 3.278 | 7.125 | 3.314 | 7.250 | 6.251 |
| *P. quadrilobata* | 1.250 | 0.620 | 0.125 | 0.125 | 0.000 | 0.000 | 0.000 | 0.000 |
| *P. ligni* | 0.250 | 0.250 | 3.750 | 1.820 | 2.875 | 3.834 | 1.875 | 1.356 |
| *E. longa* | 0.000 | 0.000 | 0.625 | 0.375 | 0.125 | 0.354 | 0.000 | 0.000 |
| *S. filicornis* | 0.000 | 0.000 | 0.000 | 0.000 | 0.625 | 1.061 | 0.625 | 0.744 |
| *C. torquata* | 0.000 | 0.000 | 0.750 | 0.620 | 0.500 | 0.756 | 0.375 | 0.744 |
| *S. fragilis* | 0.000 | 0.000 | 0.000 | 0.000 | 0.375 | 0.744 | 0.125 | 0.354 |
| *P. gouldie* | 0.125 | 0.125 | 0.625 | 0.375 | 0.250 | 0.463 | 0.375 | 0.744 |
