# Supplementary material for: Community disruption in small biogenic habitats: A coastal invader overcomes habitat complexity to alter community structure
Source: PLoS One. 2020 Oct 26;15(10):e0241116. doi: 10.1371/journal.pone.0241116 (PMC7588051; doi:10.1371/journal.pone.0241116)
Supplement: S4 Table — Column at the left identifies the comparisons and the average dissimilarity. (DOCX) [file pone.0241116.s005.docx]

**S4 Table. Ranking of 5 species contributing the most to the dissimilarity between green crab enclosures (GC) and the other three treatments in the field experiment: mud crab enclosure (MC), Control cage (CC) and no cage (NC), up to a minimum of 60% dissimilarity).** Column at the left identifies the comparisons and the average dissimilarity.

| **Comparison** | **Species** | **Average densities** | | **Dissimilarity** | |
| --- | --- | --- | --- | --- | --- |
|  |  | **GC** | **Other** | **Average (%)** | **Cumulative (%)** |
| GC vs MC | *H. filiformis* | 2.75 | 12.38 | 15.63 | 21.62 |
| 72.30% diss. | *M. arenaria* | 1.50 | 6.13 | 9.03 | 34.10 |
|  | *G. gemma* | 4.25 | 5.63 | 8.88 | 46.39 |
|  | *N.succinea* | 0.75 | 5.25 | 7.26 | 56.43 |
|  | *G. oceanicus* | 2.75 | 2.00 | 6.18 | 64.98 |
|  |  |  |  |  |  |
| GC vs CC | *G. gemma* | 4.25 | 8.88 | 14.57 | 20.64 |
| 70.61% diss. | *H. filiformis* | 2.75 | 6.50 | 8.42 | 32.56 |
|  | *G. oceanicus* | 2.75 | 4.75 | 8.13 | 44.08 |
|  | *N. succinea* | 0.75 | 3.88 | 6.34 | 53.06 |
|  | *A. rubricata* | 1.5 | 4.38 | 5.62 | 61.02 |
|  |  |  |  |  |  |
| GC vs NC | *H. filiformis* | 2.75 | 7.25 | 12.28 | 17.99 |
| 68.26% diss. | *G. gemma* | 4.25 | 6.75 | 12.23 | 35.91 |
|  | *M. arenaria* | 1.50 | 6.00 | 11.13 | 52.22 |
|  | *G. oceanicus* | 2.75 | 0.63 | 4.82 | 59.29 |
|  | *N. succinea* | 0.75 | 2.63 | 4.55 | 65.95 |
